# Seventy Years Ago

**Published:** 1916-01

**Authors:** 


### SEVENTY YEARS AGO

### Abstracts From Buffalo Medical Journal, 1846.

**Vaccination After Exposure to Small Pox.** Danger of Humanized Virus. Dr. Samuel Salisbury, Jr., Boston M. & S. Jour., mentions a woman who sat for half an hour at the bedside of a friend with small-pox. She went immediately to a physician and was vaccinated. (Evidently lay propaganda were not unknown in those days). The course of the vaccine disease was regular, save for considerable fever and constitutional symptoms on the 8th and 9th days but she developed severe small-pox on the 18th day. Not knowing of her exposure, virus taken from her arm on the 8th day, was used by another physician for vaccinating 2 adults and 5 children. Of the adults it is known only that they did not develop small-pox. None of the 5 children had been vaccinated previously and all had a typic vaccination. Three, however, subsequently had mild small-pox. Dr. Salisbury holds that the small-pox was communicated by vaccination and not by accidental conveyance of infection. (While the use of humanized virus is no longer necessary or desirable, there remains the live issue, not only with regard to variola but to infections generally, whether prophylactic vaccination should be performed after exposure. Many of the older authorities advocated this practice, under the conception that the variola and vaccinia, so to speak, ran a race, in which the milder infection might win. The editor, presumably Dr. Austin Flint 1st, himself, cites two cases of vaccination after exposure, which protected against small-pox, or, at least, in which small-pox failed to develop).

**New Test for Bile and Sugar.** This is the well known Pettenkoffer test abstracted from Annalen der Chem. & Pharm. To a little of the suspected liquid in a test tube, 2/3 its volume of strong sulphuric acid is added drop by drop to avoid excessive heat which would decompose choleic acid. 2-5 drops of 5 parts of sugar to 4 of water are added and the mixture shaken. A violet red color indicates bile. The sulphuric acid must be free from sulphurous acid and the temperature must remain under 144 F. The reversal of the test is suggested for diabetic urine.

**Physicians in London.** Mitchell's Directory is quoted as listing 2,157; 330 physicians, 245 surgeons, 1,582 general practitioners.

**Arsenical Antidote.** It is stated that the hydrated peroxid of iron acts only when uncombined acids of arsenic have been taken and does not fix Fowler's solution. The peracetate, with excess of peroxid of iron is recommended. M. Duplas, Phil. Mag.

---

**Pain in Operations.** Alf. A. M. Velpeau, New Elements of Operative Surgery, 1st Am. Ed. (Book Review). "To avoid pain in operations is a chimera. Neither opium, lettus, hellebore nor rue are any more capable to 'lenify the pain' than infusions of Iapis or the sympathetic powders of Digby."

---

**Entrance of Air Into Veins.** Of 40 cases analysed by Velpeau, this accident was considered probable in 10, uncertain in 8, extremely probable in 2, almost certain in 3. The rest could not be judged from the details reported.

---

**Beginning of the A. M. A.** The National Medical Convention was held on May 5, 1846, at the University Medical College, New York, agreeably to appointment by the State (of N. Y.) Medical Society. About 100 delegates, representing 16 states (there were then 29, including Florida, Texas and Iowa, recently admitted) were present. Committees were appointed to report to the next meeting in Philadelphia, May, 1847, and the session adjourned at the end of two days.

---

**Latin Prescriptions are Considered Absurd.** "The sooner the profession is stripped of all remnants of affectation and mystery, the better."

---

**Dr. F. H. Hamilton's Series on An European Tour,** mentions seeing the collection of Surgeon's instruments and the surgeon's house, at Pompeii; also the druggist's house and baths.

---

**A Case of Extrauterine Foetation** in a woman of 32 who had had one child 10 years previously, is reported by Drs. M. Bristol, attendant and A. S. Sprague, operating surgeon. Operation was performed but the patient died of septic peritonitis.

---

**Atropine.** According to Novak, atropine is very effective in the treatment of dysmenorrhea. In the Wiener Klinische Wochenschrift, he reports 38 cases treated with this drug, in 30 of which the pain was reduced to a negligible quantity, or cured completely, with atropine. He gives the drug by mouth or rectum.

